# MET-EGFR dimerization in lung adenocarcinoma is dependent on EGFR mtations and altered by MET kinase inhibition

**DOI:** 10.1371/journal.pone.0170798

**Published:** 2017-01-31

**Authors:** Elena Ortiz-Zapater, Richard W. Lee, William Owen, Gregory Weitsman, Gilbert Fruhwirth, Robert G. Dunn, Michael J. Neat, Frank McCaughan, Peter Parker, Tony Ng, George Santis

**Affiliations:** 1 Division of Asthma, Allergy and Lung Biology, King's College London, Guy's Hospital, London, United Kingdom; 2 Richard Dimbleby Department of Cancer Research, Randall Division of Cell and Molecular Biophysics, King’s College London, Guy's Medical School Campus, London, United Kingdom; 3 Department of Imaging Chemistry and Biology, Division of Imaging Science and Biomedical Engineering, King’s College London, The Rayne Institute/St. Thomas' Hospital, London, United Kingdom; 4 Department of Cancer Genetics, Viapath, Guy’s and St Thomas’ NHS Foundation Trust, Guy's Hospital, London, United Kingdom; 5 Division of Cancer Studies, King’s College London, Guy's Medical School Campus, London, United Kingdom; 6 Protein Phosphorylation Laboratory, Francis Crick Institute, London, United Kingdom; 7 Breast Cancer Research Unit, King’s College London, Guy's Hospital, London, United Kingdom; 8 UCL Cancer Institute, Paul O' Gorman Building, University College London, London, United Kingdom; Universita degli Studi di Parma, ITALY

## Abstract

Advanced lung cancer has poor survival with few therapies. EGFR tyrosine kinase inhibitors (TKIs) have high response rates in patients with activating *EGFR* mutations, but acquired resistance is inevitable. Acquisition of the *EGFR* T790M mutation causes over 50% of resistance; MET amplification is also common. Preclinical data suggest synergy between MET and EGFR inhibitors. We hypothesized that EGFR-MET dimerization determines response to MET inhibition, depending on *EGFR* mutation status, independently of MET copy number. We tested this hypothesis by generating isogenic cell lines from NCI-H1975 cells, which co-express L858R and T790M *EGFR* mutations, namely H1975^L858R/T790M^ (EGFR TKI resistant); H1975^L858R^ (sensitized) and H1975^WT^ (wild-type). We assessed cell proliferation *in vitro* and tumor growth/stroma formation in derived xenograft models in response to a MET TKI (SGX523) and correlated with EGFR-MET dimerization assessed by Förster Resonance Energy Transfer (FRET). SGX523 significantly reduced H1975^L858R/T790M^ cell proliferation, xenograft tumor growth and decreased ERK phosphorylation. The same was not seen in H1975^L858R^ or H1975^WT^ cells. SGX523 only reduced stroma formation in H1975^L858R^. SGX523 reduced EGFR-MET dimerization in H1975^L858R/T790M^ but induced dimer formation in H1975^L858R^ with no effect in H1975^WT^. Our data suggests that MET inhibition by SGX523 and EGFR-MET heterodimerisation are determined by *EGFR* genotype. As tumor behaviour is modulated by this interaction, this could determine treatment efficacy.

## Introduction

Epidermal growth factor receptor (EGFR) tyrosine kinase inhibitors (EGFR-TKIs) have revolutionised treatment of non-small cell lung cancer (NSCLC) in patients with *EGFR* mutations. These mutations cause constitutive kinase activity and are oncogenic drivers in 10–20% of Caucasian patients and up to 50% of eastern Asians.[1] Such mutations induce conformational changes in the receptor that alter the dimerization interface, destabilize the inactive state and increase kinase activity to 50 times that of the wild type (WT) EGFR.[2] The *EGFR* exon 21 L858R and in-frame exon 19 deletions account for 85% of such mutations.[3] Whilst responses are often impressive, resistance is inevitable. The commonest mechanism for resistance is acquisition or clonal expansion of the *EGFR* exon 20 T790M mutation.

Amplification of the MET receptor represents an important alternative resistance mechanism [4, 5, 6, 7]. MET is a high affinity tyrosine kinase receptor for hepatocyte growth factor (HGF).[8] Derailment of normal MET signaling is associated with invasive growth, tumor progression and metastases; [9] aberrant MET signaling can result from MET over-expression, amplification or mutations, all of which are relevant in NSCLC.[4, 5, 6, 7] MET amplification predicts worse survival in NSCLC, [10] it has been implicated in 5–20% of patients with acquired resistance to EGFR TKI [11, 12, 13, 14] and correlates with response to MET inhibitor therapy [13]. Blockade of MET is a therapeutic strategy in EGFR TKI resistance. The most advanced agents, METMAb, a MET neutralizing antibody and Tivantinib, a small molecule inhibitor of MET have both failed in phase III clinical trials [15]; despite this, there is considerable interest in the therapeutic potential of MET inhibition in NSCLC. In fact, Crizotinib, a MET proto-oncogene, receptor tyrosine kinase (MET) tyrosine kinase inhibitor (TKI) is currently in clinical trial showing good results for both MET amplification and MET exon 14 skipping [14].

MET may exert its oncogenic effects through crosstalk with other membrane receptors including the EGFR family, as evidenced by MET and EGFR co-expression in lung cancer cell lines, [16] crosstalk between EGFR and MET signaling pathways and direct co-immunoprecipitation.[16, 17, 18] Moreover, MET amplification in association with *EGFR* mutations additionally has a worse clinical prognosis than *EGFR* mutations alone.[10] In light of these observations, we sought to understand the importance of EGFR and MET interaction and we have hypothesized that the efficacy of MET inhibition can be influenced by *EGFR* mutation status. We explored this hypothesis by evaluating the response of three lung adenocarcinoma cell lines that differ only in their *EGFR* genotype to the MET inhibitor SGX523 *in vitro* and in a murine xenograft model derived from the same cells. Our data suggest that EGFR mutations can determine the effect of MET inhibition independently of MET copy number, by changing EGFR-MET dimerisation. As tumor behaviour is modulated by this interaction, this could determine treatment efficacy.

## Results

### EGFR-MET interaction is modulated by *EGFR* mutations

**Fig 1:**
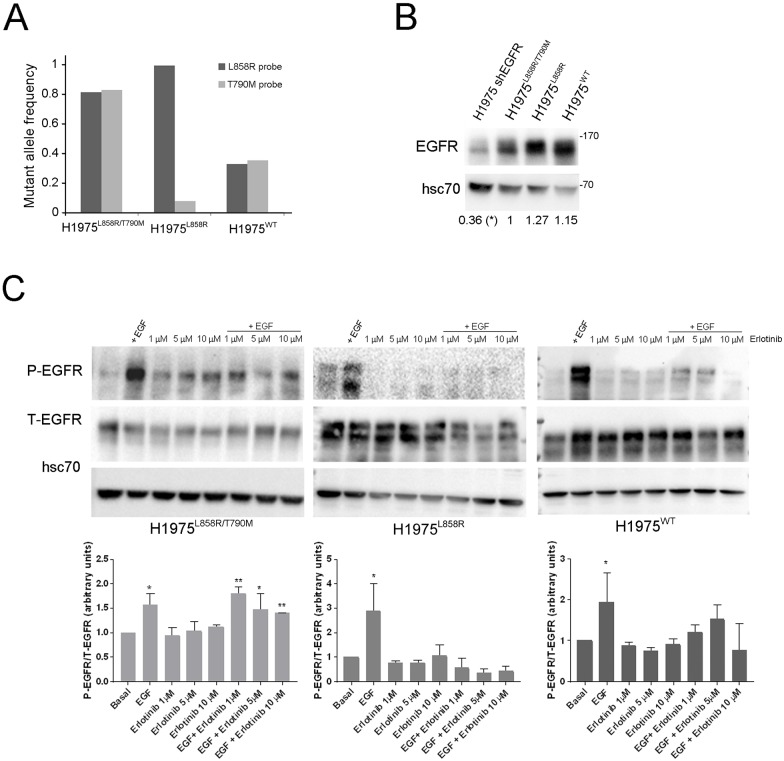
*In vitro* validation of the H1975 EGFR mutant cell lines. (A) Relative mutant allele frequency was compared in cDNA from each cell line by Digital droplet PCR. (B) WB of total EGFR levels in the H1975^L858R/T790M^ cell line before and after lentivirus infection with a shEGFR and in the H1975 cell lines. hsc70 levels are shown as loading control. Values beneath blots are relative levels of T-EGFR compared to the H1975^L858R/T790M^ cell line from 2 independent experiments (C) WB of phospho and total EGFR in H1975 derivative cell lines untreated or treated with EGF (100ng/mL) for 15 min, Erlotinib (1μM, 5μM or 10 μM) for 1 hour or both. hsc70 levels were used as loading control. Quantification of the WB is shown above the figure from 3 independent experiments. Error bars is SD (*p<0.05 and **p<0.001).

To assess if EGFR-MET interaction is modified by *EGFR* mutations, we first generated two novel cell lines by modification of the NCI-H1975 lung adenocarcinoma cell line that harbours L858R and T790M (L858R/T790M) mutant EGFR (to be referred to from here on as H1975^L858R/T790M^). We used lentiviral shRNA knockdown of EGFR (targeting the 5’ UTR of EGFR) in the H1975^L858R/T790M^, followed by transfection with a plasmid encoding wild/type (wt) *EGFR* and *EGFR* with the L858R mutation, to generate the H1975^WT^ and the H1975^L858R^ cell lines respectively. Relative allele frequency (*L858R vs*. *T790M*) in these cell lines was quantified by digital droplet PCR. We observed equal *L858R* and *T790M* copies in the H1975^L858R/T790M^ cells and a clear reduction of L858R and T790M *EGFR* alleles in the H1975^WT^ cells, confirming their effective knockdown following shEGFR treatment; we also observed decrease of the EGFR-T790M allele frequency in the H1975^L858R^ cells (Fig 1A). Using Western blot (WB), we showed the total levels of EGFR in the generated cell lines (Fig 1B) and that the H1975^L858R^ and H1975^WT^ cells became sensitive to the EGFR TKI Erlotinib upon removal of the T790M *EGFR* sequence even at a low concentration of Erlotinib (Fig 1C). Introduction of a GFP plasmid in the H1975^L858R/T790M^ cell line didn’t affect the Erlotinib resistance (Fig A in S1 File).

**Fig 2:**
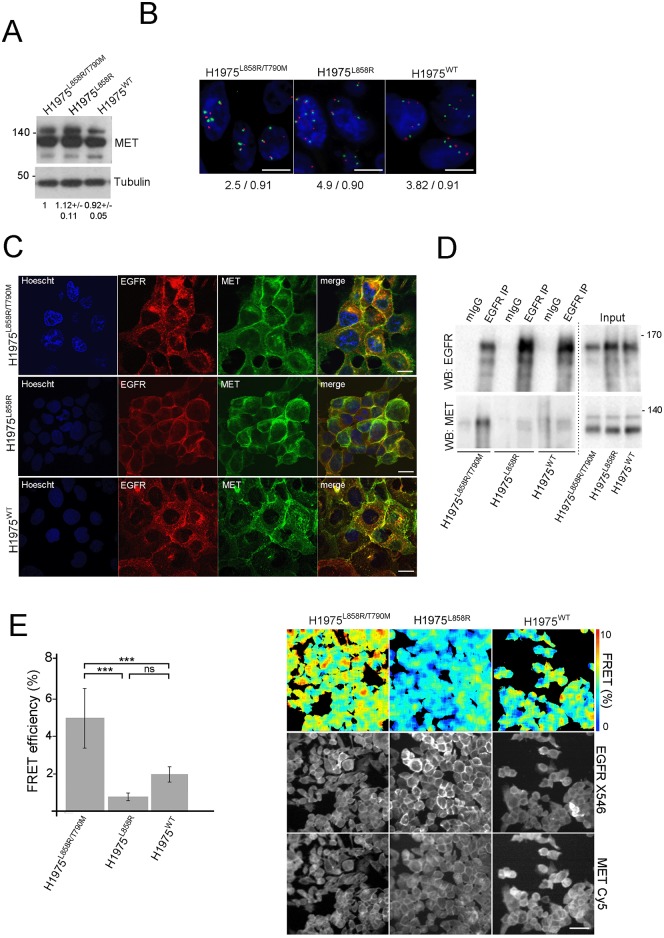
EGFR-MET interaction is modulated by EGFR mutations. (A) Western blot (WB) of total MET levels in H1975 derived cells. Tubulin levels are shown as loading control. Values beneath blots are relative levels of MET compared to the total levels in the H1975^L858R/T790M^ cell line from 2 independent experiments +/−SD (B) MET (7q31) (red signal) copy number analysis by FISH in the three H1975 cell lines using the Leica Kreatech C-MET (7q31)/SE7 FISH probe (KBI-10719). The green signal indicates the chromosome 7 centromere control probe. Scale bar 10 mm. Average copy number and ratio between MET and chromosome 7 centromere probe are also indicated (n = 30 cells). (C) Immunofluorescence of total EGFR (Alexa546 –red in the image) and MET (Cyanine 5 –green in the image) in H1975 derived cells. Hoescht dye was used to stain the nuclei of the cells. Merge panels are also shown. Bars, 20 μm. (D) Co-immunoprecipitation (IP) of EGFR in H1975 derived cell lines. The EGFR antibody was used to immunoprecipitate. EGFR and MET levels are shown in both bound and input fractions. The gels shown in the figure were run separately for the bound and input fractions, as indicated by the dotted line, under the same experimental conditions. (E) Fluorescence lifetime imaging was performed on cells plated to sub-confluence on cover-slips and time-resolved analysis in Tri2. Quantification of average FRET efficiency (*** p < 0.0005) is shown, as well as representative pseudocolour lifetime images showing FRET efficiency and corresponding grayscale donor (EGFR-Alexa 546) and acceptor (MET-Cyanine 5) intensity images. Scale bar: 50μm.

We then explored the interaction between EGFR and MET in the cells lines studied. We observed no differences in basal MET protein levels between the cell lines (Fig 2A). Given that MET expression is not a good marker for MET activation we also treated the three cell lines with HGF for different times to asses for the phospho-MET levels. We could observe no difference in the activity of MET in the different cell lines (Fig B in S1 File). As *MET* amplification has been implicated as a mechanism by which secondary resistance EGFR TKI can emerge in NSCLC, we used a MET/cep7 fluorescence *in situ* hybridization (FISH) probe and calculated the ratio of MET to chromosome 7 centromere signals and the mean copy number of MET. The MET/cep7 ratio was <1 in all three cell lines; however, MET copy number was increased in all (Fig 2B).

Immunofluorescence staining and confocal images showed that both proteins co-localise similarly in the membrane of the three cell types (Fig 2C). Immunoprecipitation result showed there was more interaction between EGFR and MET in H1975^L858R/T790M^ compared to H1975^L858R^ and H1975^WT^ cells (Fig 2D), which provides evidence of potential differential interaction between MET and different EGFR mutant types. Since a direct protein interaction (nanometer (nm) proximity) between MET and EGFR cannot be inferred by these experiments, we assessed Förster Resonance Energy Transfer (FRET) using fluorescence lifetime imaging microscopy (FLIM) which is the gold standard technique for measuring protein proximity within the typically <10nm range [19, 20, 21, 22]. Results show that highest FRET efficiency between MET and EGFR occurred in H1975^L858R/T790M^ cells, in contrast with significantly lower FRET efficiency in the H1975^L858R^ and H1975^WT^ cells (Fig 2E). These results demonstrate that EGFR and MET can interact directly at the cell membrane and that the level of interaction is significantly higher in H1975^L858R/T790M^ compared to H1975^L858R^ and H1975^WT^ cells.

### Inhibition of MET changes the EGFR-MET interaction both *in vitro* and *in vivo*

**Fig 3:**
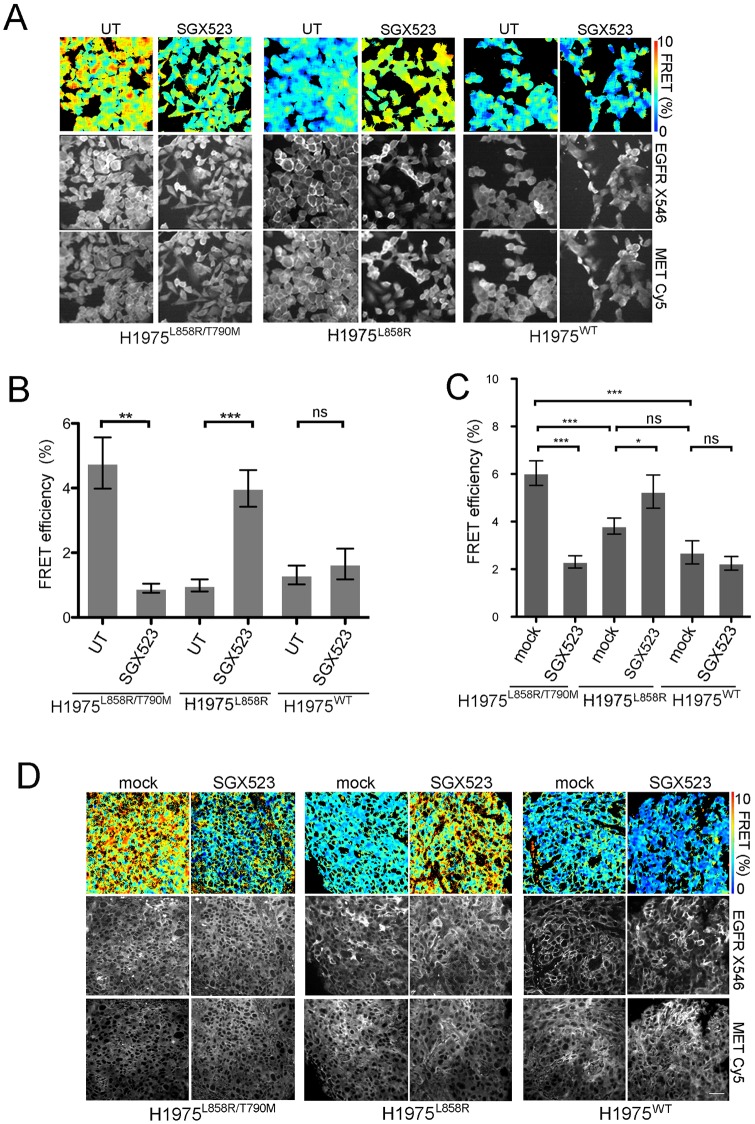
Inhibition of MET changes the EGFR-MET interaction both *in vitro* and *in vivo*. (A) Fluorescence lifetime imaging performed in the three H1975 cell lines plated on coverslips with or without treatment with SGX523 (5 μM) for 24 hours. Representative pseudocolour lifetime images showing FRET efficiency accompanied by corresponding grayscale donor (EGFR-Alexa 546) and acceptor (MET-Cyanine 5) intensity images are shown. Scale bar: 50μm. (B) Bar graph showing quantification for average FRET efficiency in the three cell lines with or without SGX523 treatment (*** p < 0.0005, ** p = 0.001). (C) Quantification of average FRET of EGFR-MET interaction performed in xenograft tumors from each H1975 cell line in mice receiving mock or SGX523 treatment (60 mg/kg) for 12 days (*** p<0.001, * p<0.05). (D) Representative lifetime images for EGFR:MET FRET in xenograft tumors accompanied by corresponding grayscale donor (EGFR-Alexa 546) and acceptor (MET-Cyanine 5) intensity images. Scale bar, 50μm.

In view of the observed differences in the interaction between EGFR and MET in our cells, we hypothesised that MET inhibition would also result in different outcomes. To test this, we used SGX523, a selective MET kinase inhibitor.[23] After confirming rapid (15min) and sustained (48hr) SGX523 inhibition of MET phosphorylation in the three cell lines (Fig C in S1 File), we measured change in the interaction between MET and EGFR using FRET before and after MET inhibition by SGX523. We found that the interaction between MET and EGFR in H1975^L858R/T790M^ cells was significantly reduced in the presence of SGX523. By contrast, SGX523 led to a significant increase in MET-EGFR interaction compared to baseline in H1975^L858R^ cells (Fig 3A and 3B). There was no significant difference in the FRET between MET and EGFR before and after MET inhibition in the H1975^WT^ cells. These results provide evidence to support the hypothesis that MET inhibition by SGX523 altered the direct interaction between MET and EGFR in a mutation-specific manner in these cells.

**Fig 4:**
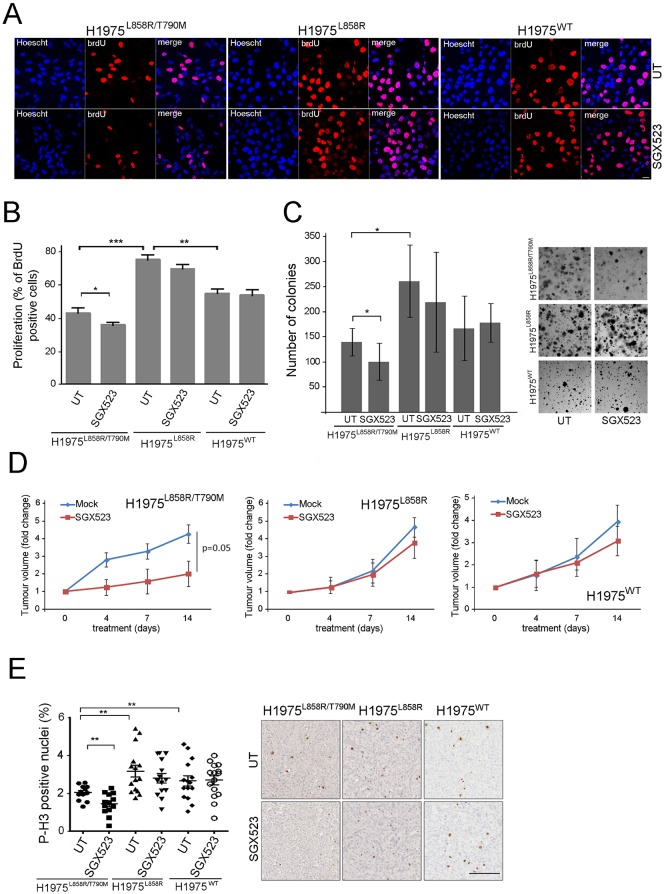
*In vitro* and *in vivo* effects of MET inhibition on cell proliferation. (A) Representative images of the three cell lines grown at 60% confluence on coverslips and treated or not with SGX523 (5 μM) for 24 hours. Scale bar, 20μm. The BrdU positive nuclei (red) show the cycling cells, and the Hoechst dye was used to stain all the nuclei of the cells (in blue). (B) Quantification of the proliferation rates as described in (A) (*p <0.04, **p<0.005, ***p<0.0001). (C) Soft agar colony formation in the H1975 derivate cell lines. The graph shows the number of colonies after 3 weeks of growing in the presence or absence of SGX523 (5 μM) (*p <0.001). Representative images of untreated and SGX523 treated colonies are also shown. (D) Graphs showing the fold change in volumes in 16 xenografts tumors (8 coming from mice subjected to vehicle and 8 from SGX523 treatment) coming from the three H1975 cell lines during the 12 days of treatment. Tumor volumes were measured at the indicated times using a calliper and calculated in based of the equation 0.4xAxB^2 (A, the long axis and B, the short axis of the tumor). (E) Quantification of phospho-histone H3 (P-H3) staining in the xenograft derived tissue in the presence or absence of SGX523 (**p<0.05) Representative images are also shown. Bar, 250 nm.

To test whether the differential effects of SGX523 on MET and EGFR interaction could be reproduced *in vivo*, we generated a xenograft model using the H1975-derived mutant cell lines. H1975^L858R/T790M^, H1975^L858R^ and H1975^WT^ cells were injected into the flanks of BalbC mice and tumors were allowed to grow for 2 weeks. Mice were then subjected to 12 days of treatment with SGX523 or vehicle administered daily by oral gavage. Tumor volumes were measured every two days until animals were culled and tissue collected (see Fig 4D). WB and immunohistochemistry (IHC) (Fig D (i) and (ii) in S1 File) confirmed target tissue drug delivery.

We observed the highest FRET between MET and EGFR in H1975^L858R/T790M^ derived tumors, and this was again significantly reduced by SGX523 (Fig 3C and 3D). In H1975^L858R^ derived tumors, MET-EGFR interaction at baseline was low and increased significantly by SGX523. There was no significant change in FRET between MET and EGFR in xenograft tissue derived from H1975^WT^ cells. These results mirror the *in-vitro* findings with these cells (Fig 3C and 3D). We conclude that MET interacts with EGFR differently in cells that encode WT, L858R and L858R/T790M-EGFR and SGX523 modifies this interaction in opposite directions in L858R and L858R/T790M-EGFR encoding cells *in vitro* and *in vivo*.

### *In vitro* and *in vivo* effects of MET inhibition on cell proliferation

In order to assess functional consequence of MET kinase inhibition, we assessed the proliferation rate of the generated cells before and after MET inhibition. We found that the H1975^L858R^ cells were more proliferative than the H1975^L858R/T790M^ or H1975^WT^ cells (Fig E (i) and (ii) in S1 File). MET inhibition significantly decreased proliferation rate in H1975^L858R/T790M^ cells only (Fig 4A and 4B). We also assessed proliferation in a 3D environment using an anchorage independent growth assay. As expected, we found that the H1975^L858R^ cells produced most colonies (Fig E (iii) in S1 File). In the presence of SGX523, there was a significant decrease in the number of large colonies in H1975^L858R/T790M^ cells. SGX523 had no effect in the 2D or 3D proliferation rate in the case of the H1975^WT^ cells or in the H1975^L858R^ cells (Fig 4C).

We then analysed the *in vivo* effect of oral administration of SGX523 on tumor growth and proliferation. As was the case *in vitro*, the H1975^L858R^ was the most proliferative cell line *in vivo* (Fig 4D and Fig F (i) in S1 File). The H1975^L858R/T790M^-derived tumors in SGX523 treated mice grew more slowly than in vehicle treated mice; this effect was evident after 4 days of SGX523 administration and was sustained up to 14 days. SGX523 had no effect in the proliferation in the H1975^L858R^ and H1975^WT^ derived tumors over the 14-day growth period (Fig 4D). This result was confirmed using the proliferation marker phospho-Histone H3 (Fig 4E).

### *In vitro* and *in vivo* effects of MET inhibition on stroma remodeling

**Fig 5:**
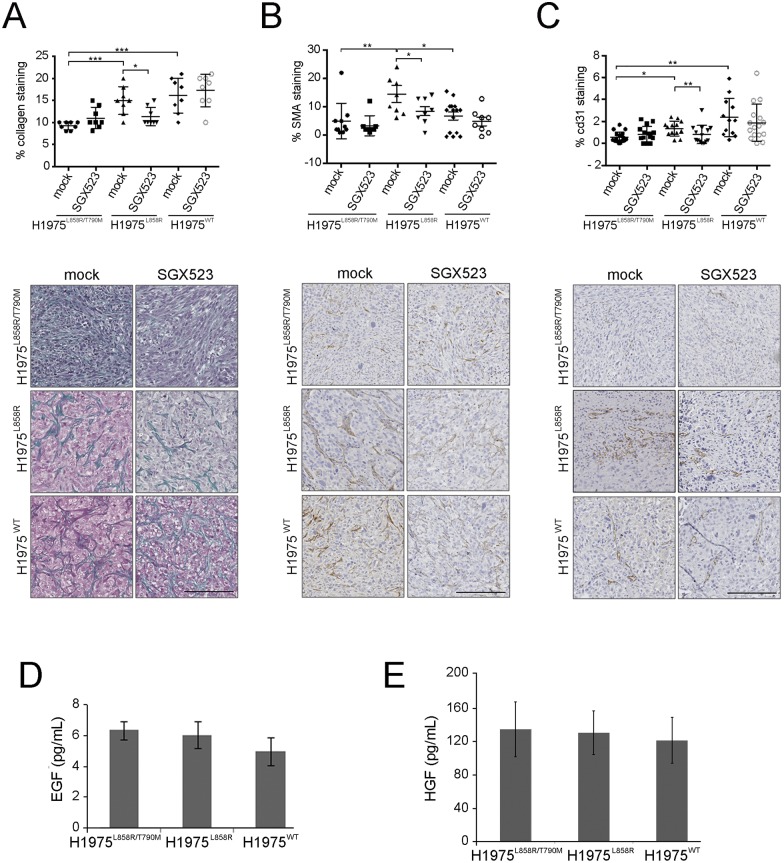
*In vitro* and *in vivo* effects of MET inhibition on stroma remodeling. Quantification and representative images of (A) collagen, (B) α-SMA and (C) cd31 staining of xenografts tumors (FFPE) grown from each H1975 derivate cell line coming from mice treated or not (mock) with SGX523 (60 mg/kg). Bar 250 nm. Quantification of the staining is shown above the images and was performed using Image J. (*p<0.05, **p<0.01, ***p<0.001). EGF (D) and HGF (E) quantification assessed by ELISA in each H1975 derivate cell line supernatants.

We observed marked morphological differences in the xenograft tumors derived from the different cell lines. H1975^L858R/T790M^-derived tumors were dense and cellular with tightly packed cells oriented in similar directions. The H1975^L858R^-derived tumors had abundant intra-tumoral stromal tissue deposition leading to highly segregated cells that appeared to grow by interaction with the stroma (Fig F (ii) in S1 File). Given this different tumours appearance, as well as the important role proposed for MET in tumour-tissue interaction and in cell migration/metastasis, we analysed stromal compartment markers, namely collagen deposition (Masson’s trichrome), anti-smooth muscle actin staining (α-SMA), a marker of activated fibroblasts and cd31 staining to assess for neovascularization, before and after SGX523 treatment. The H1975^L858R^-derived tumors demonstrated the most substantial collagen deposition and α-SMA staining (Fig F (iii) in S1 File). We also found that H1975^WT^ and H1975^L858R^-derived-tumors showed more angiogenesis than the H1975^L858R/T790M^-derived tumors (Fig F (iii) in S1 File). When we compared the untreated *vs*. the SGX523 treated tumors, we observed significant reduction in collagen (MT) (Fig 5A), α-SMA (Fig 5B) and cd31 (Fig 5C) staining in the H1975^L858R^. SGX523 had no effect on these parameters in H1975^L858R/T790M^ and H1975^WT^ derived tumors.

These results suggest a paracrine response to MET inhibition only in H1975^L858R^ cells. We dismiss a different autocrine response, as EGF and HGF secreted by the three cell lines was similar (Fig 5D).

### *In vitro* and *in vivo* effects of MET inhibition on ERK, AKT and FAK phosphorylation

**Fig 6:**
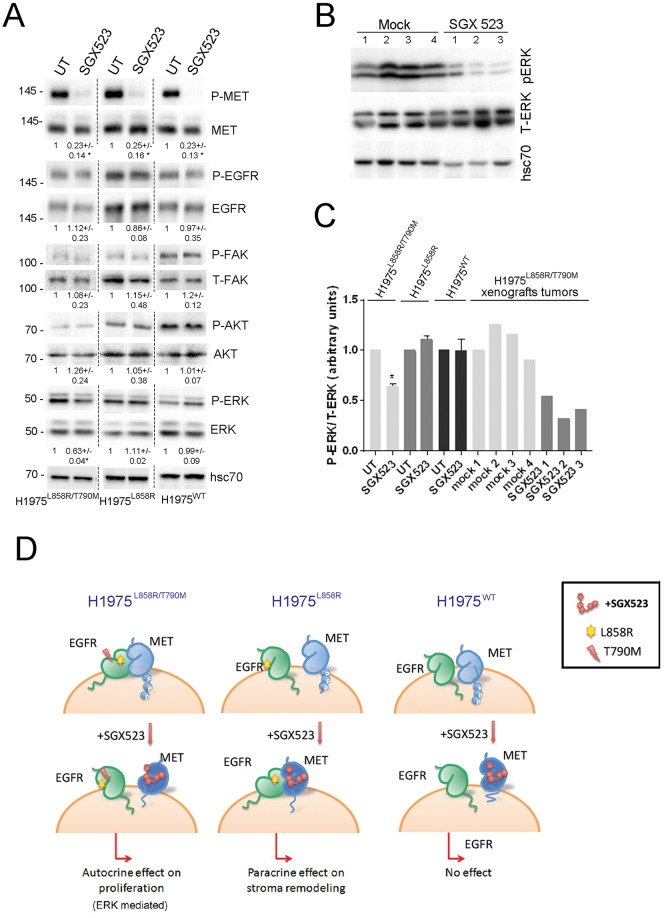
*In vitro* and *in vivo* effects of MET inhibition on ERK, AKT and FAK phosphorylation. (A) WB of phospho- and total EGFR, MET, AKT, ERK and FAK from cell lysates of the different H1975 cell lines treated or not with SGX523 (5 μM) for 24 hours. Gels shown are representative of three experiments, and were run separately for each cell line, as indicated by the dotted line, under the same experimental conditions. Hsc70 levels are also shown as loading control. Values beneath blots are relative levels of each phospho vs total protein comparing the untreated (UT) and the SGX523 treated conditions for each cell line for 3 independent experiments and for 2 independent experiments in the case of the P-FAK/FAK +/−SD (*p<0.05, **p<0.001) (B) WB of phospho- and total-ERK in the extracts coming from the H1975^L858R/T790M^-derived xenografts tumors in vehicle (mock) and SGX523 treated mice. hsc70 is also shown as loading control. (C) Quantification of the P-ERK vs T-ERK WBs shown in (A) and in (B) (*p<0.05). (D) Schematic model demonstrating effect of SGX523 on EGFR-MET dimerization pattern and consequences for tumor characteristics following treatment.

We finally investigated whether there were also differential effects of MET kinase inhibition on signaling pathways. We treated H1975^L858R/T790M^, H1975^L858R^ and H1975^WT^ cells with SGX523 for 24 hours. Phosphorylation of MET was inhibited but we saw no change in the phosphorylation of EGFR with the SGX523 treatment. We then assessed phosphorylation levels of ERK, AKT and FAK after SGX523. The only significant finding from these studies was a reduction in ERK phosphorylation in SGX523 treated H1975^L858R/T790M^ cells (Fig 6A and 6C). We repeated these experiments in the presence of EGF and or HGF in the cells treated or not with SGX523 to better understand the decrease in the stromal markers in the H1975^L858R^ but we observed no effect in the phosphorylation of AKT or FAK (Fig G in S1 File). Finally, the analysis of xenograft tumors obtained from SGX523 or vehicle treated animals also showed reduction in phosphorylated ERK in H1975^L858R/T790M^-derived xenografts (Fig 6B and 6C). These observations provide potential explanation for our observation that SGX523 had anti-proliferative effect in H1975^L858R/T790M^-derived tumors but not H1975^L858R^ and H1975^WT^ cells and tumors.

## Discussion

In this study we have demonstrated that the responses to MET inhibition in an *in vitro* and *in vivo* lung cancer model differed depending on the *EGFR* genotype. Using as a starting point the H1975 lung adenocarcinoma cell line that harbours double mutated (L858R/T790M double mutant) *EGFR*, we have developed a cellular model to minimise the contribution of confounding influences. Using Droplet PCR, WB and EGFR TKI sensitivity, we established that in each line the dominant *EGFR* genotype was of greatest functional importance. We considered the merits of a CRISPR approach to achieve purer cell populations. However, the validation steps described and the tumour cell heterogeneity inherent to our approach could offer a good reflection of the clinical situation.

Our key novel finding from *in vitro* and *in vivo* studies is that MET inhibition by SGX523 reduced proliferation and tumor growth in the H1975^L858R/T790M^ cells and inhibited indices of stromal deposition in H1975^L858R^ cells. MET inhibition in H1975^L858R/T790M^ cells also suppressed ERK signaling consistent with interference with an autocrine signaling loop. Conversely, in H1975^L858R^ cells the effects of SGX523 behavior was more consistent with a paracrine activity of the MET receptor (see Fig 5D). These variations in SGX523 responses in each cell line led us to hypothesize that these differences could be in part a product of the interaction between the EGFR and MET.

Existing evidence supports EGFR-MET crosstalk. Our own data showing co-localization and co-immunoprecipitation of EGFR and MET at the cell membrane is in agreement with these observations. Importantly, receptor dimerisation is essential for both MET and EGFR signaling and intracellular effect. EGFR can dimerise with itself and other members of the HER family. Previous studies have shown that cell proliferation and tumorigenesis are enhanced in tumor xenografts co-expressing HER family heterodimers compared to those expressing single receptors.[24] For example, the well-characterized HER2-HER3 pair is the most oncogenic in breast cancer.[25] It has also been suggested that MET can interact not only with EGFR but with other proteins that drive receptor activation, such as integrins, [26] plexin B1, [27] and CD44v6.[28] The relevance of any such interactions in the context of different *EGFR* genotypes and in response to MET inhibition has not been explored.

To the best of our knowledge ours is the first study to utilise FRET FLIM imaging to demonstrate EGFR-MET direct dimerisation. Importantly, we observed that the FRET between EGFR and MET differed according to *EGFR* genotype, providing a potential novel mechanism by which responses to MET inhibition can differ. In H1975^L858R/T790M^ cells, SGX523 reduced EGFR-MET FRET. Conversely FRET was increased by SGX523 in H1975^L858R^ cells but had no significant effect on FRET in H1975^WT^ cells. SGX523 is known to preferentially bind the less active, unphosphorylated form of MET.[23] Therefore, the differences observed suggest that EGFR^L858R/T790M^ favours the phosphorylated form of MET; by contrast, EGFR^L858R^ only dimerised with MET in the presence of SGX523, which suggests the opposite. No such modulation was seen in H1975^WT^ suggesting that the altered binding by MET in its active or inactive form was related to the presence of mutated and not WT-EGFR. Altered dimerisation between MET and mutant EGFR in EGFR^L858R/T790M^ and H1975^L858R^ cells in the presence of SGX523 could be explained by SGX523-induced conformational changes; such conformational changes following treatment with small molecule kinase inhibitors are recognized and can be associated with unexpected responses to treatment. For example HER2-HER3 dimer formation occurs in breast cancer following treatment with Lapatinib.[29] Our studies cannot discriminate between the binding of MET with an EGFR monomer or homodimer. Dimer formation may activate one or other of the receptors or indeed both. In the case of higher order oligomers, MET may facilitate or impede EGFR homodimer formation or modulate the kinase activity of EGFR molecules within the homodimer. EGFR remained phosphorylated after treatment with SGX523 (Fig 5A). Whether MET is additionally capable of phosphorylating EGFR or modulating EGFR activity allosterically is not clear.

We observed clear phenotypic differences between the three cell lines. This may result from different tendency to activation or affinity for substrate. For example L858R and L858R/T790M have increased ligand-activated signaling activity compared with WT-EGFR, particularly with respect to downstream effectors involved in cell survival and activation of transcription.[30] This is in agreement with published work showing that the *EGFR* mutants mediate significantly increased ligand-independent activation of the receptor.[31] This could explain that the presence of *EGFR* L858R mutant increases proliferation in the cells and tumors, and the novel finding that these cells also produce tumors with higher stromal markers. Alternatively, the EGFR-MET dimer offers a novel interpretation of the differences between the lines. In the H1975^L858R/T790M^ cells, where the EGFR-MET dimer is present at baseline, downstream signals may be driven by this heterodimer. The addition of SGX523 resulted in loss of EGFR-MET heterodimer and reduction in phosphorylated ERK in the H1975^L858R/T790M^ cells and derived tumours, in agreement with this hypothesis. In the cells expressing EGFR L858R there is no EGFR-MET dimer at baseline. The addition of SGX523 increases the dimerisation between the receptors, which resulted in the decrease of stromal markers such as % of collagen, SMA or cd31 staining, in the absence of inhibition in any of the typical signaling pathways analysed. However, as previously suggested, the interplay of EGFR with MET could provide a secondary set of effectors molecules through MET binding sites and furthermore through other receptors with which MET is reported to interact, such as HER3.[13] It is therefore likely that additional as yet unidentified pathway is active whilst the AKT/FAK pathway may not dominate in MET inhibition in this model.

What is the implication of our findings on the potential role of MET inhibition in lung cancer? Current emphasis is on the role of MET amplification as biomarker to select patients for MET inhibitor therapy in both treated or untreated NSCLC.[15, 16, 32, 33, 34]. Our observations that the effects of MET inhibition are influenced by EGFR-MET dimerisation and in turn *EGFR* genotype, suggest novel mechanisms to understand how best to target MET in NSCLC and highlight the importance of understanding the interaction between multiple driver mutations when facing treatment resistance. Designing a FRET-FLIM assay that measures EGFR-MET heterodimerisation could potentially play a role in patient selection for MET targeted therapy. It is also interesting to speculate how our observations on the effects of SGX523, a MET kinase inhibitor, may influence treatment options in NSCLC. Whether it would be preferable in the future to target MET with a monoclonal antibody or through direct targeting of its kinase domain is unknown. A better understanding of crosstalk between therapeutic targets, which could be targeted in parallel, may also be of benefit to treatment efficacy and cost-effective use.

In summary, we have demonstrated using FRET that we can measure the interaction between EGFR and MET and we have made the important observation that response to MET inhibition correlates with EGFR-MET dimerisation, which is influenced by *EGFR* genotype. This could result from the different EGFR-MET dimers that arise following treatment with SGX523. The next important steps will be to determine how this information can be used to select effective therapies targeting MET.

## Materials and methods

### Reagents and antibodies

Recombinant Human Epidermal Growth Factor (EGF) and Hepatocyte Growth Factor (HGF) were purchased from Peprotech. SGX523 was purchased from GE and Erlotinib from Cayman chemicals; both were solubilised in DMSO. All antibodies (Abs) were purchased from commercial sources as indicated. For tissue and cell staining, Phospho-Histone H3 was from Millipore, α-SMA from AnaSpec Inc., cd31 from Abcam, total cMET (D1C2) from Cell Signaling Technology and EGFR Ab from Novocastra/Leica. For immunoprecipitation, the EGFR Ab was from Santa Cruz Biotechnology (sc-120). For Western Blot, phospho-EGFR (Y1173), total EGFR phospho-MET (Y1234), total MET, phospho-ERK (Thr202/Tyr204), total ERK, phopho-AKT (Ser473), total AKT, phosphor-FAK (Tyr397) and total FAK were from Cell Signaling Technology; tubulin was from Millipore and hsc70 from Santa Cruz Biotechnology. For proliferation assays, anti-Brdu was purchased from Abcam.

### Plasmid construction

Plasmid encoding non-tagged pcDNA3-EGFR was a kingly gift from Dr. Tai Kiuchi (Tohoku University, Aoba-ku, Sendai, Miyagi, Japan). For control transfections, pEGFP-N3 vector from Clontech was used. Plasmid for L858R EGFR mutant was created by site-directed mutagenesis, using the following forward primer: GTC AAG ATC ACA GAT TTT GGG **cgg** GCC AAA CTG CTG GGT GCG and reverse primer: CGC ACC CAG CAG TTT GGC **ccg** CCC AAA ATC TGT GAT CTT GAC. The reaction was made using a site-directed mutagenesis kit from Stratagene and following manufacturer’s instructions.

### Cell lines, cell culture and transfection

All cell lines were cultured at 37°C and 5% CO_2_. Phoenix Ampho HEK293T cells were maintained in Dulbecco´s modified Eagle medium and the NCI-H1975 cells lines in RPMI-1640 (Invitrogen), both mediums with 10% fetal bovine serum (FBS, Gibco). To generate H1975^L858R^ and H1975^WT^ cells, shRNA against 5’ UTR EGFR GFP tagged was obtained from Sigma (siMission) and used to produce lentiviral particles in HEK293T cell line. Stable cells expressing shEGFR-GFP were selected with puromycin (1.5 μg/ml) for 5 days. After selection, pcDNA3 constructs containing WT or L858R *EGFR* were transfected in the cells with Fugene HD following manufacturer’s instructions. Selection was performed for 5 days with G418 (50 μg/ml). EGFR total, L858R EGFR, and L858R + T790M EGFR levels were analysed by WB and Droplet PCR. For experiments requiring growth factor stimulation serum-starved cells were treated with 100 ng/ml EGF or 250 ng/ml HGF.

### Droplet polymerase chain reaction

Cells were plated for confluence and DNA extracted from cells using a DNeasy Blood and Tissue Kit (Qiagen) according to manufacturer’s protocol. Digital Droplet PCR was performed using reference primers/probe sets validated on the BioRAD QX100 mdPCR system (Biorad) using commercially available reference standards. Data was analysed using Quantasoft (Biorad) software. Droplets were scored as positive or negative based on relative fluorescence intensity in FAM or VIC/HEX channels.

### Western blotting (WB)

Cells were lysed in buffer (0.05 M Tris-HCl, 0.15 M NaCl, 1% Triton X-100, pH 7.2), containing protease and phosphatase inhibitors. After centrifugation, proteins in the supernatant were quantified, boiled with Laemmli buffer, resolved by SDS-PAGE and transferred to a nitrocellulose membrane. WB was performed using standard procedures.

### Immunofluorescence microscopy

Cells cultured on cover slips were fixed using 2% PFA in PBS for 15 min and then permeabilised with 0.25% Triton X-100. After blocking with 1% BSA and 1% FBS in PBS, cells were incubated with the primary antibodies ON at 4°C. Cells were then washed and incubated with secondary antibodies (all from Jackson ImmunoResearch Laboratory). Confocal images were obtained with an LSM510 microscope (Carl Zeiss) and analysed using LSM Viewer software.

### Fluorescence *in situ* hybridization (FISH)

Cells were plated on coverslips and fixed with Carnoy’s fixative (3:1 methanol to acetic acid). After washing with PBS, incubation with the MET/cep7 FISH probe was performed for 16 hours at 36°C after a 5minute denaturation at 72°C. The coverslips were washed in 0.4xSSC at 72°C for 5 minutes, followed by 2 minutes in 4xSSC/Tween and two rounds of 2 minutes in PBS at room temperature. The coverslips were then mounted onto slides with DAPI counterstain and analysed using a fluorescence microscope.

### Immunoprecipitation

Cells were lysed in lysis buffer (50 mM Tris-HCl pH 7.4, 150 mM NaCl, 1 mM EDTA, 1 mM EGTA, 10% glycerol, 1% Triton X-100, 10 mM NaF, 1 mM Na_3_VO_4_, 10 mM N-ethylmaleimide, 0.01 μM Calyculin A) with Protease inhibitor cocktail set I (Roche). After centrifugation, the supernatants were incubated overnight at 4°C with anti-EGFR or an irrelevant IgG, and subsequently for an additional hour with protein A/G-Agarose beads (Alpha Diagnostic International Inc.). After centrifugation, the immunoprecipitates were washed and subjected to SDS-PAGE and analysed by WB.

### BrdU incorporation

Cell proliferation was determined by 5’-Bromo-Uridine (BrdU) based assay. Cells were plated for sub-confluence on coverslips in a 24-well plate and BrdU was added to the cells for 3 hours. Coverslips were then prepared for immunofluorescence staining as described below with an additional 15 minutes DNA denaturation step using 1.5M Hydrochloric Acid (HCl) and using a BrdU Ab. Hoechst was used to stain all the cell nuclei. Images were obtained LSM510 microscope (Carl Zeiss) and quantified in ImageJ using a macro to automate cell counting for red vs blue nuclei.

### Soft-agar growth assay

Anchorage-independent growth was evaluated as described before.[35] Briefly, 1x10^4^ cells were plated in complete DMEM containing 0.3% soft agar in 6-cm plates over a solidified DMEM plated in containing 0.7% soft agar layer. Medium was added twice a week to maintain humidity. After 3 weeks, colonies were stained with crystal violet (0.02%) for 1 hour and counted.

### Wound healing experiments

Cells were seeded to confluence. A horizontal wound was made through the middle of the wells using a micropipette tip. Cells were allow to migrate (heal the wound) for different times (0, 2, 8, 20 and 28 hours). Images were taken and the wound closure was quantified using Image J software analysis.

### *In vivo* tumorigenicity assay

H1975^WT^, H1975^L858R^ and H1975^L858R/T790M^ cell lines (3x10^6^) were injected subcutaneously into the two posterior flanks of BALB/c nude mice (Charles River Laboratories). For each cell line, 16 female 5 weeks old mice were used. Mice were followed weekly and tumors allowed growing for 13 days after injection. Tumors were measured with a caliper in long and short axes and volume was determined in based of the equation 0.4xAxB^2 (A, the long axis and B, the short axis of the tumor). At day 14^th^ after injection mice were divided randomly in two groups (6 animals/group) and vehicle or SGX523 (drug treatment) was started. 60 mg/kg of SGX523 (equilibrated suspensions in 0.5% Methocell A4M) was administered for 12 days, daily, by oral gavage. At day 25^th^ after injection mice were culled using CO_2_ and tumours were removed aseptically with dissecting scissors and weighed. All animals were maintained under specific pathogen-free conditions and handled in accordance with the Institutional Committees on Animal Welfare of the UK Home Office (The Home Office Animals Scientific Procedures Act, 1986). All animal experiments were approved by the Ethical Review Process Committee at King’s College London and carried out under license from the Home Office, UK.

### Tissue samples, immunohistochemistry and histopathological analysis

Tissue samples obtained from xenografts were fixed with formalin and paraffin-embedded. Immunohistochemical analyses were performed using 3 μm sections. Briefly, slides were dewaxed and antigen retrieval was performed using 0.1M citrate pH 6.0 buffer at 120°C for 10 min, followed by blocking in TBS-Tween 0.1% + 1% BSA + 1% FBS. Primary antibodies were added overnight at 4°C. As secondary antibodies, peroxidase-conjugated (Envision+) anti-rabbit and anti-mouse Ig reagents from Dako were used for 1h. Non-immune (Santa Cruz Biotechnology) or pre-immune rabbit serum was used as negative controls. Reactions were developed using diaminobenzidine (DAB) as chromogenic substrate. Images from digitalized scans of the glass slide specimens were obtained at magnification ×20 (0.45 μm/pixel resolution) using a Hamamatsu Nanozoomer 2.0 HT. All quantifications were done using Image J.

### FRET determination by FLIM measurements

Coverslips or FFPE tissue was prepared as described as above for immunofluorescence except for the added step of quenching of autofluorescence by 15 minutes immersion in NaBH_4_ (1 mg/mL). FLIM was performed using time-correlated single-photon counting (TCSPC) with a multiphoton microscope system as described previously.[19] Analysis was performed in Tri2 for time resolved analysis. To analyze FRET in tissue xenografts samples, a dedicated algorithm was used that masks autofluorescent lifetime measurements to reveal true FRET.[20]

### Enzyme-linked immunosorbent assay (ELISA)

Cells were plated in 6 well plates and incubated overnight. Supernatant was collected and EGF and HGF ligands were measured by sandwich enzyme-linked immunosorbent assay manufacturer’s instructions.

### Statistical analysis

Student t test for parametric data, with ANOVA comparison for more than two groups, determined statistical differences. Probability of p<0.05 was considered significant. Graphs were prepared in Excel or in Prism (GraphPad) software.

## Supporting information

S1 File (DOCX)
