# Supplementary material for: MET-EGFR dimerization in lung adenocarcinoma is dependent on EGFR mtations and altered by MET kinase inhibition
Source: PLoS One. 2017 Jan 31;12(1):e0170798. doi: 10.1371/journal.pone.0170798 (PMC5283661; doi:10.1371/journal.pone.0170798)
Supplement: S1 File — (DOCX) [file pone.0170798.s001.docx]

**Supporting information**


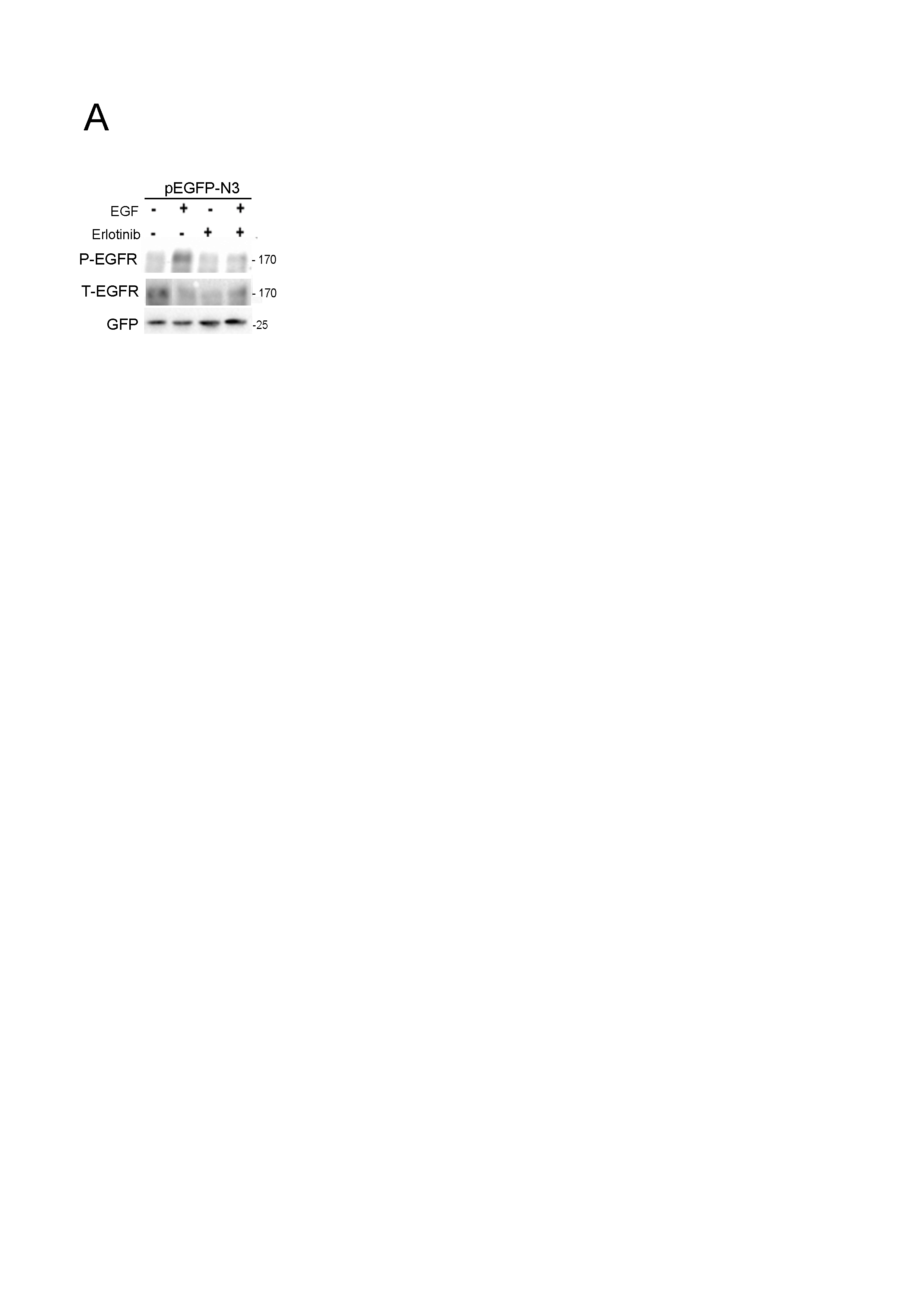


**Figure A in S1 File:** WB of phospho and total EGFR in the H1975^L858R/T790M^ cell line transfected with a PEGFR-N3 empty vector and treated with EGF (100ng/mL) for 15 min, Erlotinib (1μM) for 1 hour or both. GFP levels were used as loading control.

**
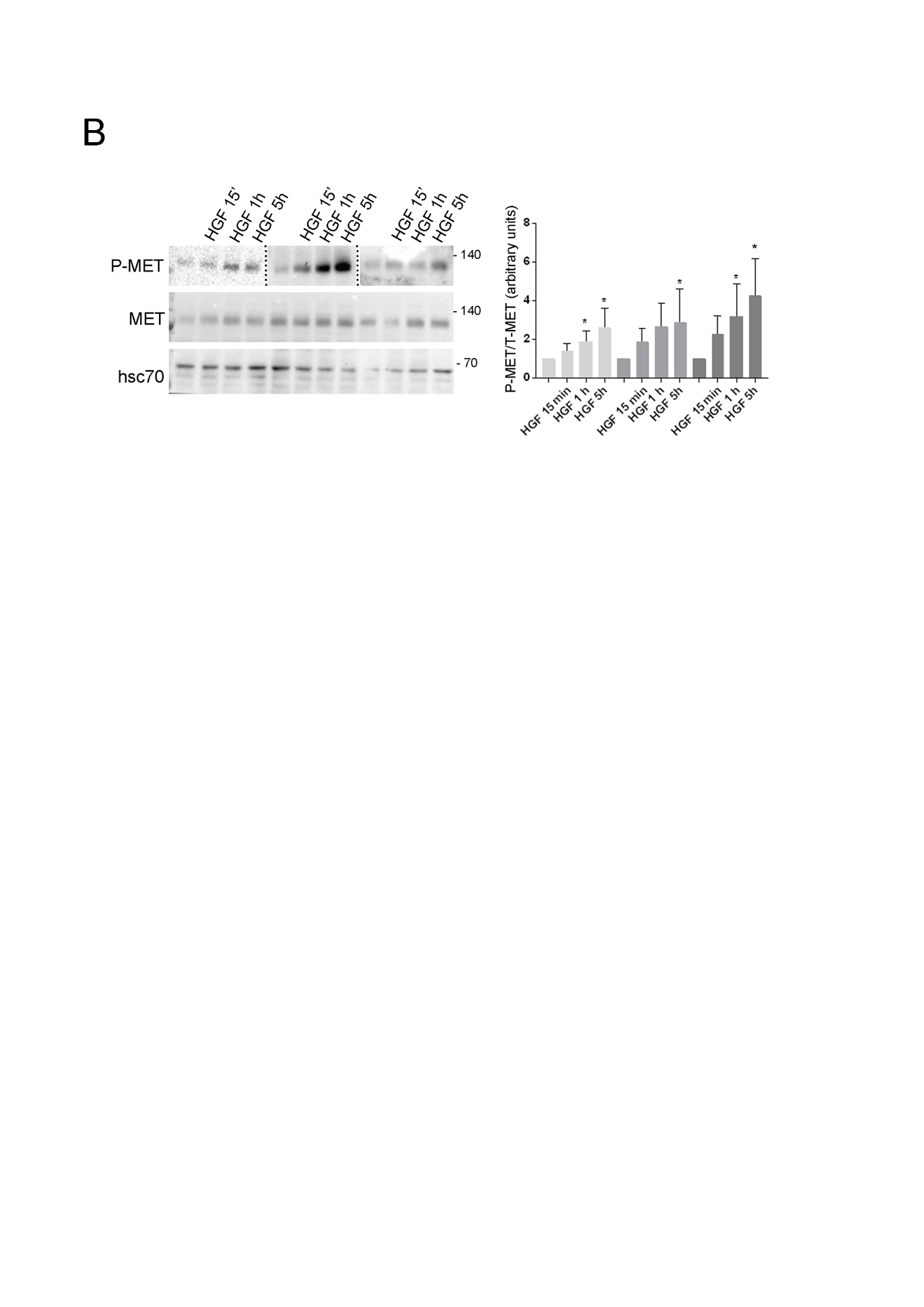
Figure B in S1 File:** WBs of phospho and total MET in cell lysates from untreated H1975 derivative cells and in cells treated with HGF (25 ng/mL) for the indicated times. A graph showing the relative quantification for each cells line compared to the untreated cells is shown. Hsc70 is shown as loading control.


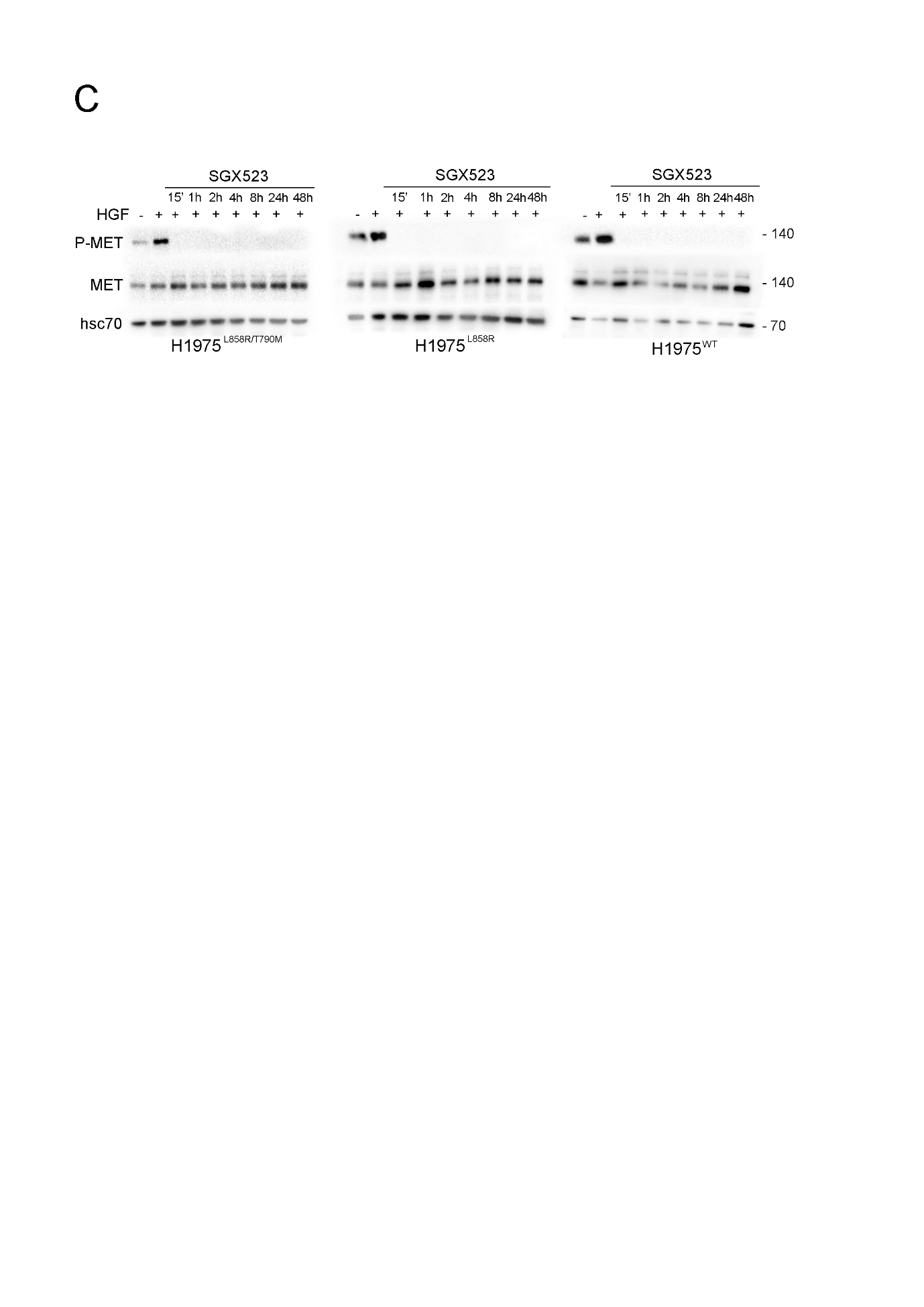


**Figure C in S1 File:** WBs of phospho and total MET in cell lysates from untreated H1975 derivative cells, treated with HGF (25ng/mL) for 15 min ± pretreatment with SGX523 (5 μM) for the indicated times. hsc70 was used as loading control.


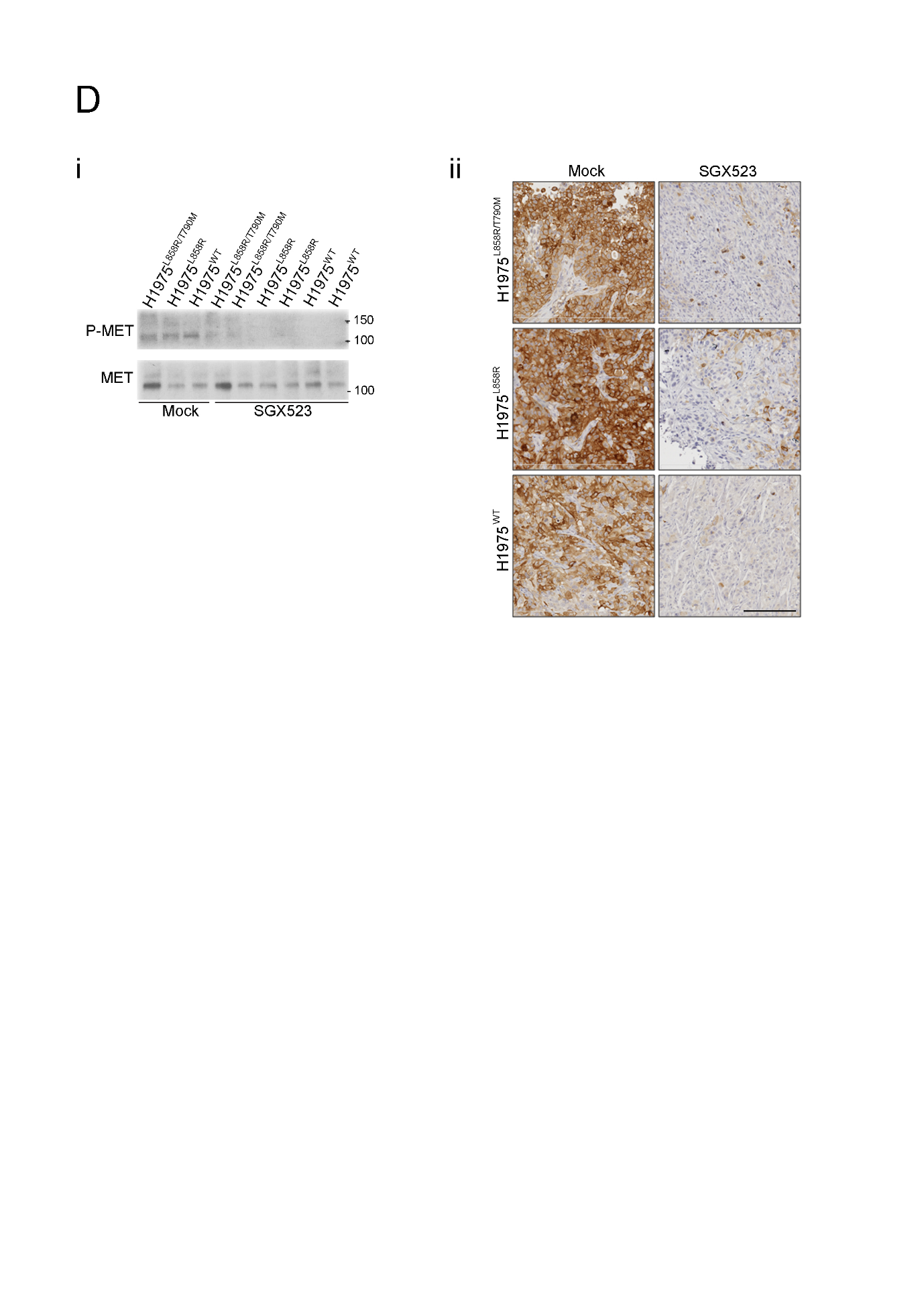


**Figure D in S1 File:** (i) WB of phospho and total MET in xenograft tumor lysates grown from each of the H1975 cell lines. Vehicle (mock) or SGX523 treated mice (60mg/kg) were used as indicated. (ii) Representative images of phospho-MET staining in xenografts tumors grown from each H1975 derivate cell line coming from mice treated with vehicle (mock) or SGX523 as indicated. Bar, 250 nm.

**
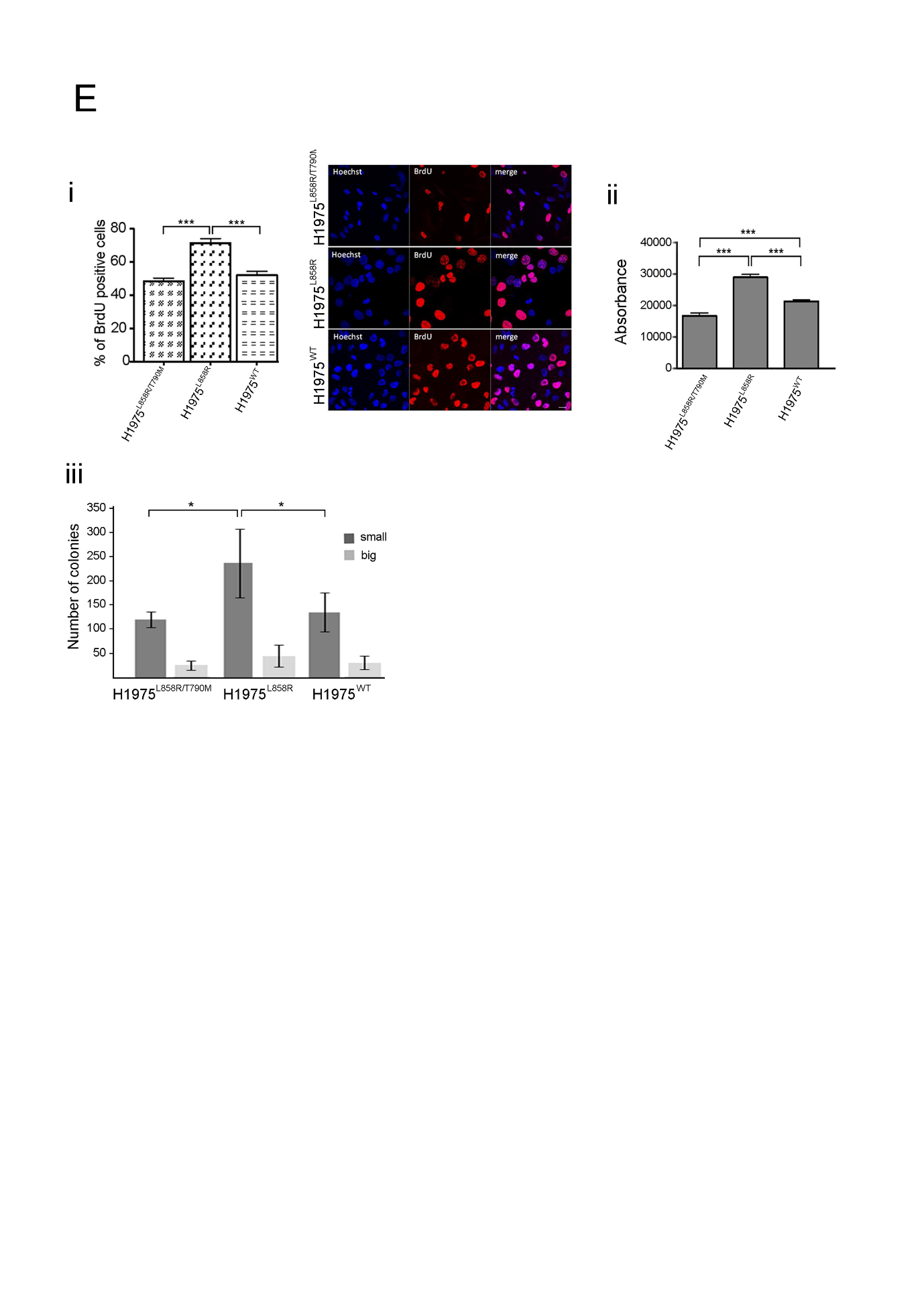
Figure E in S1 File:** (i) Quantification of the proliferation rates measured by BrdU incorporation in the three H1975 cell lines (p *** < 0.005). Representative images of the three cell lines grown at 60% confluence on coverslips and allow to grow for 24 hours. The BrdU positive nuclei (red) show the cycling cells, and the Hoechst dye was used to stain all the nuclei of the cells (in blue). Scale bar, 20μm. (ii) Quantification of the proliferation rates measured using Prestoblue Cell Viability reagent (p *** < 0.005). (iii) Soft agar colony formation in the H1975 derivate cell lines. The graph shows the number of colonies after 3 weeks of growth. Small colonies were defined as 100-1500 μm circularity, and large colonies, more 1500 circularity, and counted using Image J.

**
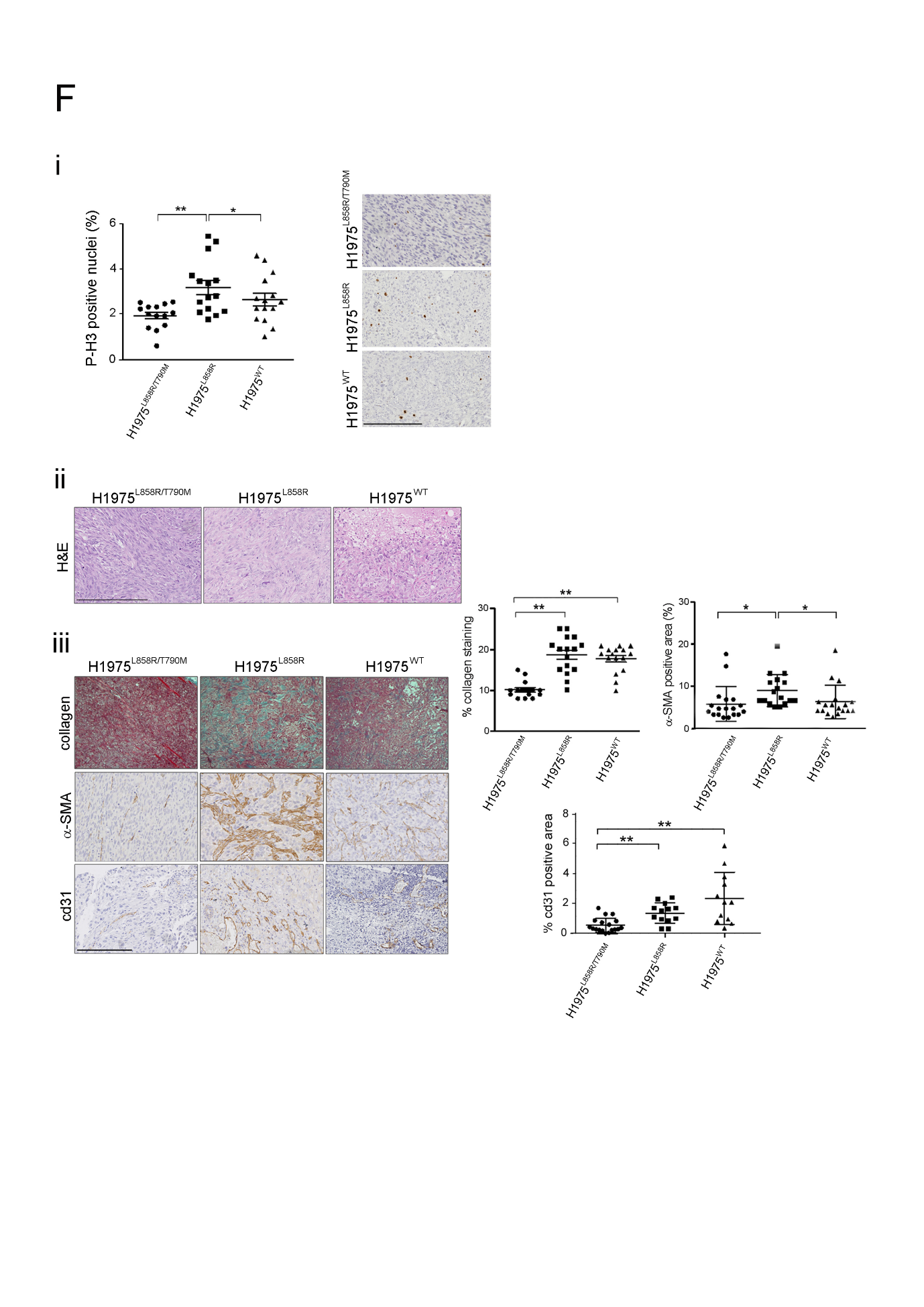
Figure F in S1 File:** (i) Quantification of phosphor-Histone3 (P-H3) positive nuclei in the xenografts tumors coming from each of the H1975 cell lines (*p<0.05, **p<0.001). Representative images of the P-H3 staining are shown. (ii) Haematoxylin and eosin (H&E) staining of xenografts tumors (FFPE) grown from each H1975 derivate cell line. Bar, 400 mm. (iii) Representative images of collagen, α-SMA and cd31 staining of xenografts tumors (FFPE) grown from each H1975 derivate cell line. Bar, 400 mm. Quantification of the staining was performed using Image J and graphs were obtained using GraphPrism (*p<0.05, **p<0.001).

**
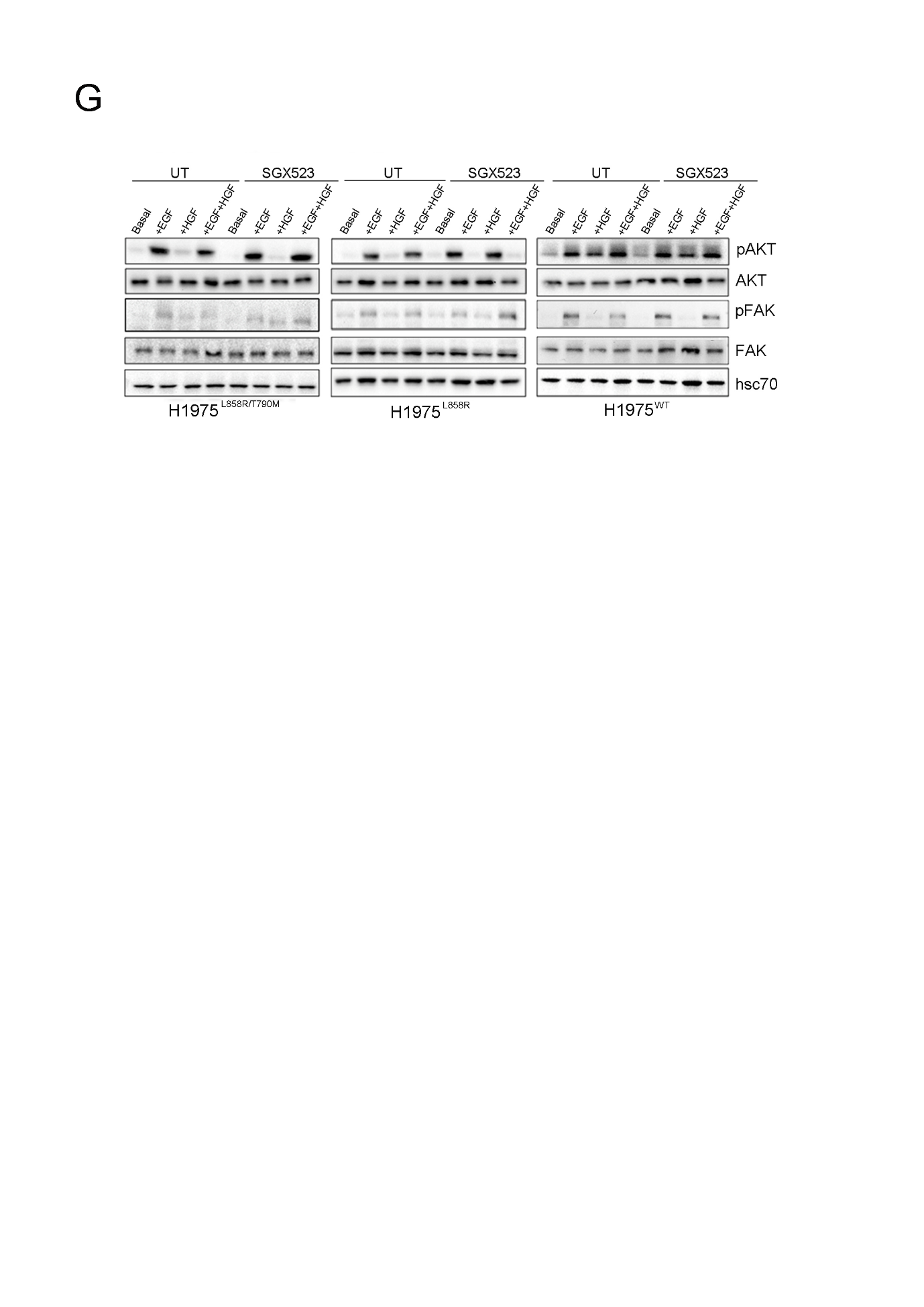
Figure G in S1 File:** WB of phospho and total AKT and FAK in cell lysates from the H1975 derivative cells untreated or treated with SGX523 for 1 hour and with HGF (25ng/mL), EGF (100 ng/mL) or both for 15 min. hsc70 levels were used as loading control.
